# Diabetes impairs the vascular effects of aldosterone mediated by G protein-coupled estrogen receptor activation

**DOI:** 10.3389/fphar.2015.00034

**Published:** 2015-03-02

**Authors:** Nathanne S. Ferreira, Stêfany B. A. Cau, Marcondes A. B. Silva, Carla P. Manzato, Fabíola L. A. C. Mestriner, Takayuki Matsumoto, Fernando S. Carneiro, Rita C. Tostes

**Affiliations:** ^1^Department of Pharmacology, Ribeirao Preto Medical School, University of São PauloSão Paulo Brazil; ^2^Department of Pharmacology, Biological Sciences Institute, Federal University of Minas GeraisBelo Horizonte, Brazil; ^3^Department of Physiology and Morphology, Institute of Medicinal Chemistry, Hoshi UniversityTokyo, Japan

**Keywords:** diabetes, aldosterone, mineralocorticoid receptor, G protein-coupled estrogen receptor, mesenteric arteries

## Abstract

Aldosterone promotes non-genomic effects in endothelial and vascular smooth muscle cells via activation of mineralocorticoid receptors (MR) and G protein-coupled estrogen receptors (GPER). GPER activation is associated with beneficial/protective effects in the vasculature. Considering that vascular dysfunction plays a major role in diabetes-associated complications, we hypothesized that the beneficial effects mediated by vascular GPER activation, in response to aldosterone, are decreased in diabetes. Mesenteric resistance arteries from female, 14–16 weeks-old, control and diabetic (*db/db*) mice were used. Phenylephrine (PhE)-induced contractions were greater in arteries from *db/db* vs. control mice. Aldosterone (10 nM) increased maximal contractile responses to PhE in arteries from control mice, an effect elicited via activation of GPER. Although aldosterone did not increase PhE responses in arteries from *db/db* mice, blockade of GPER, and MR decreased PhE-induced contractile responses in *db/db* mesenteric arteries. Aldosterone also reduced the potency of acetylcholine (ACh)-induced relaxation in arteries from both control and *db/db* mice via MR-dependent mechanisms. GPER antagonism further decreased ACh-induced relaxation in the control group, but did not affect ACh responses in the diabetic group. Aldosterone increased extracellular signal-regulated kinase 1/2 phosphorylation in arteries from control and *db/db* mice by a GPER-dependent mechanism. GPER, but not MR, gene, and protein expression, determined by RT-PCR and immunoblotting/immunofluorescence assays, respectively, were increased in arteries from *db/db* mice vs. control arteries. These findings indicate that aldosterone activates both vascular MR and GPER and that the beneficial effects of GPER activation are decreased in arteries from diabetic animals. Our results further elucidate the mechanisms by which aldosterone influences vascular function and contributes to vascular dysfunction in diabetes. Financial Support: FAPESP, CNPq, and CAPES, Brazil.

## INTRODUCTION

Diabetes represents a major public health challenge. In 2013, 382 million people exhibited diabetes and in 2035 this number will rise to 592 million (International Diabetes Federation [IDF], 2013). Vascular dysfunction, or endothelial dysfunction, occurs early in the pathogenesis of diabetic vasculopathy and is closely implicated in the complications associated with all forms of diabetes (De Vriese et al., 2000). In the leptin receptor deficient *db/db* mouse, a rodent model of obesity and type 2 diabetes, vascular dysfunction is characterized by impaired vasodilatation and by increased responses to vasoconstrictor stimuli (Pannirselvam et al., 2002; Guo et al., 2005).

Aldosterone, a mineralocorticoid hormone with a key role in the regulation of hydroelectrolytic balance, has important effects in the vasculature, contributing to inflammation, oxidative stress, remodeling, and vascular dysfunction in cardiovascular and metabolic diseases, including diabetes (Schiffrin, 2006; Briet and Schiffrin, 2012). Aldosterone effects are associated with genomic and non-genomic mechanisms following the activation mineralocorticoid receptors (MR; Davies and Struthers, 2002; Davies et al., 2005; Swaminathan et al., 2008). Aldosterone induces activation of different kinases, including protein kinase C (PKC), phosphatidylinositol 3-kinase (PI3K), tyrosine kinases, epidermal growth factor receptor (EGFR), insulin-like growth factor-1 receptor (IGF-1R), and mitogen-activated protein kinases (MAPKs; Christ et al., 1995; Krug et al., 2003; Liu et al., 2003; Callera et al., 2005; Ishizawa et al., 2005). These pathways are critically involved in pathophysiological processes associated with vascular inflammation and injury (Callera et al., 2011; Whaley-Connell and Sowers, 2011; Bender et al., 2013). Accordingly, MR antagonists, such as eplerenone and spironolactone, have been shown to reverse hypertension- and diabetes-associated vascular dysfunction (Swaminathan et al., 2008; Schafer et al., 2010; Briones et al., 2012).

Aldosterone also activates G protein-coupled estrogen receptors (GPER) and induces rapid vascular effects. Gros et al. (2011) showed that aldosterone activates extracellular signal-regulated kinase (ERK)1/2, myosin light chain (MLC) and induces apoptosis in smooth muscle cells of rat aorta via activation of both GPER and MR. Furthermore, in endothelial cells aldosterone activates ERK1/2 via GPER, an effect blunted by treatment with G15, a GPER antagonist (Gros et al., 2013).

G protein-coupled estrogen receptors activation induces endothelium-dependent as well as endothelium-independent vasodilatation (Yu et al., 2011) and has been shown to mediate vascular protective effects of estrogen (E2) as well as of the GPER synthetic agonist G1 (Haas et al., 2007; Meyer et al., 2010; Lindsey et al., 2011, 2013) and aldosterone (Gros et al., 2013). Of importance, the GPER synthetic agonist G1 induces concentration-response dependent dilatation in thoracic aorta of diabetic ovariectomized rats (Li et al., 2012). Considering the paucity of information on whether aldosterone effects in resistance arteries are mediated by GPER activation and if these effects are altered in pathological conditions, such as diabetes mellitus, this study addressed the role of GPER activation on the vascular effects of aldosterone in control and *db/db* mice. We hypothesized that the beneficial vascular effects mediated by GPER activation are decreased in diabetes mellitus.

## MATERIALS AND METHODS

### ANIMALS

All experimental protocols were performed in accordance with the Guidelines for the Care and Use of Laboratory Animals published by the US National Institutes of Health (NIH Publication No. 85–23, revised 1996). Protocols were approved by the Committee for Animal Research of the Ribeirao Preto Medical School – University of Sao Paulo, Ribeirao Preto, Brazil (Protocol No. 012/2013). Fourteen to 16 weeks-old female control and *db/db* mice (purchased from The Jackson Laboratory – Bar Harbor, Maine, USA) were used. Mice were housed in individually ventilated cages (4 mice per cage – 600 cm^2^) in a room with controlled humidity (50 ± 10%) and temperature (22 ± 2°C), and light/dark cycles of 12 h. Animals had free access to food (Nuvilab mice chow pellets, Nuvital, Curitiba, Brazil) and potable tap water.

### ALDOSTERONE INCUBATION PROCEDURES

After euthanasia, mesenteric arteries (MA) were rapidly excised and cleaned from fat tissue in an ice-cold (4°C) Krebs–Henseleit-modified solution [(in mM): 130 NaCl, 4.7 KCl, 14.9 NaHCO_3_, 1.18 KH_2_PO_4_, 1.17 MgSO_4_⋅7H_2_O, 5.5 glucose, 1.56 CaCl_2_⋅2H_2_O, and 0.026 EDTA], gassed with 5% CO_2_ /95% O_2_ to maintain a pH of 7.4. Second-order branches of MA (≈2 mm in length with internal diameter ≈150–200 μm) were carefully dissected and mounted as rings in an isometric Mulvany–Halpern myograph (model 610 M; Danish Myo Technology – DMT, Copenhagen, Denmark). Changes in force were recorded by a PowerLab 8/SP data acquisition system (ADInstruments). Second-order MA were adjusted to maintain a passive force of 13,3 kPa and allowed to equilibrate for about 30 min in Krebs–Henseleit solution. After the stabilization period, arterial integrity was assessed first by stimulation of vessels with 120 mM of KCl. After washing and a new stabilization period, endothelial function was assessed by testing the relaxant effect of acetylcholine (ACh, 10 μM) on vessels contracted with phenylephrine (PhE, 3 μM). MA exhibiting a relaxant response to ACh greater than 90% were considered endothelium-intact vessels. All experiments were performed with endothelium-intact vessels.

Arterial segments were incubated with either vehicle (1% ethanol) or aldosterone (10 nM) for 30 min to verify acute effects of the hormone. This concentration was chosen based on preliminary experiments performed with different concentrations of aldosterone (100 pM–1 μM). In another set of experiments, MA were functionally evaluated after incubations for 30 min with a MR antagonist (eplerenone) or a GPER antagonist (G15) in Krebs–Henseleit solution.

### VASCULAR FUNCTION ANALYSIS

Cumulative concentration–response curves to PhE (0.1 nM–10 μM) and ACh (0.1 nM–30 μM) were performed in MA incubated with aldosterone (10 nM, for 30 min) or vehicle. To determine the involvement of MR and GPER on aldosterone effects, concentration–response curves to PhE and ACh were performed either in the absence (control) or in the presence of eplerenone (10 μM) and G15 (1 μM), respectively. Tissues were incubated with the antagonists for 30 min prior to the incubation with aldosterone. Each preparation was tested with a single agent.

### QUANTITATIVE REAL TIME PCR (RT-qPCR)

Mesenteric arteries total RNA, extracted with TRIzol (Invitrogen^®^), was treated with DNase I (1 U/μL, Promega) and used for first-strand cDNA synthesis, accordingly to the manufacturer instructions. mRNA levels were quantified in triplicate by qPCR StepOnePlus^TM^ *Life Technologies*. Specific primers for RT-qPCR were as follows: mouse MR (Nr3c2) *[Mm01241596_m1]* and GPER (Gpr30) *[Mm02620446_s1],* purchased from *TaqMan*^TM^ *– Life Technologies*. PCR cycling conditions included 10 min at 95°C, followed by 40 cycles at 95°C for 15 s, 60°C for 1 min, and 72°C for 60 s. Dissociation curve analysis confirmed that signals corresponded to unique amplicons. Specific mRNA expression levels were normalized relative to β-actin (actb) *[Mm00607939_s1]* mRNA levels using the comparative 2^ΔΔCt^ method.

### WESTERN BLOTTING

Protein expression of MR and GPER and activity of ERK1/2 protein were determined in endothelium-intact vessels by western blot analysis. After the incubation protocols were performed, vessels were frozen in liquid nitrogen and proteins were extracted, separated (40 μg) by electrophoresis on 10% polyacrylamide gels and transferred to nitrocellulose membranes. Non-specific binding sites were blocked with 1% bovine serum albumin in Tris-buffered saline solution with Tween (0.1%) for 1 h at 24°C. Membranes were incubated with antibodies (at the indicated dilutions) overnight at 4°C. Antibodies were as follows: anti-MR (1:300, Abcam), anti-GPER (1:300, Abcam), anti-ERK1/2 (1:1000, Cell Signaling), anti-phospho (pERK)1/2 (Thr202/Tyr201; 1:1000, Cell Signaling), and anti-β-actin (1:3000, Cell Signaling). After incubation with secondary antibodies, signals were revealed by chemiluminescence, visualized by autoradiography and quantified densitometrically. Results were normalized to β-actin expression and expressed as units relative to the control.

### IMMUNOFLUORESCENCE

Immunofluorescence analysis of the MR was performed following the manufacturer instructions. Formaldehyde-fixed vessels were blocked using TBS, 3% dried milk, 0.1 % Triton X-100 for 20 min at 22°C. Samples were then incubated with the primary antibody (anti-MR receptor antibody 1:200 [H10E4C9F] (ab2774) Abcam) for 1 h at 22°C, followed by incubation with the secondary antibody [anti-mouse IgG 1:500 (H + L) HSA, dyLight 549 labeled, KLP, 042-04-18-06], dilution in HEPES [4-(2-hydroxyethyl)-1-piperazineethanesulfonic acid].

For immunofluorescence analysis of the GPER, vessels were fixed with 4% paraformaldehyde for 5 min and blocked with 10% donkey serum in 0.1% PBS-0.3% TritonX for 30 min at 24°C. Samples were then incubated with primary antibody [anti-GPER 1:500 (ab39742) Abcam] in 0.3% Triton X with 0.1% PBS and 10% donkey serum for 1 h at 24°C, followed by the secondary antibody (Texas red goat anti-rabbit IgG antibody, 1:100 Vector, TI-1000), used at a 1:100 dilution in HEPES.

Nuclei were counterstained with the blue fluorescent dye 4′,6-diamidino-2-phenylindole [DAPI (1 μg/mL)]. Images were obtained using a fluorescence microscope (Automated Inverted Microscope, Leica DMI4000 B). Quantification was performed in the media and endothelial layers using the Image J program (1.47t Wayne Rasband, National Institutes of Health, USA). Same color threshold was used for analysis in all groups. The results were expressed as fluorescence intensity per area (μm^2^) relative to control intensity.

### DRUGS

Phenylephrine, ACh, aldosterone, and eplerenone were purchased from Sigma Chemical Co (St. Louis, MO, USA). G15 was purchased from Calbiochem (Poland).

### DATA ANALYSIS AND STATISTICAL PROCEDURES

Contractile responses to PhE are expressed as percentage of 120 mM KCl-induced response. No significant difference was found in 120 mM KCl contraction between arteries from control and diabetic mice (*p* > 0.05). Relaxation to ACh is expressed as percentage of 3 μM PhE-induced contraction. The individual concentration–response curves were fitted into a curve by non-linear regression analysis. *p*D_2_ (defined as the negative logarithm of the EC_50_ values) and maximal response (Emax) were compared by *t*-tests or ANOVA, when appropriated. The Prism software, version 5.0 (GraphPad Software Inc., San. Diego, CA, USA) was used to analyze these parameters as well as to fit the sigmoid curves. Data are presented as mean ± SEM. *N* represents the number of animals used. *p*-values less than 0.05 were considered significant.

## RESULTS

### ALDOSTERONE INCREASES PhE-INDUCED VASOCONSTRICTION IN CONTROL BUT NOT IN *db*/*db* MICE

**FIGURE 1:**
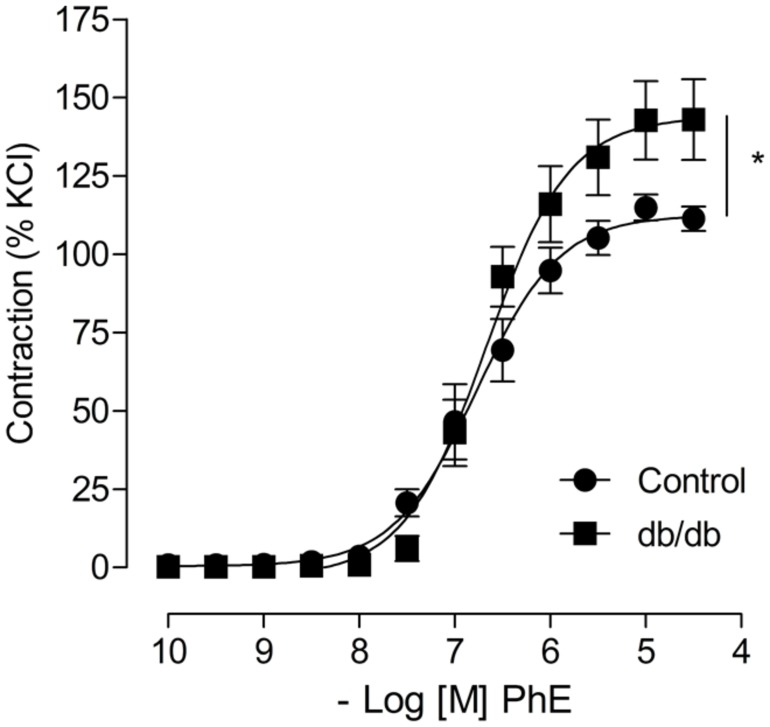
**Arteries from leptin receptor deficient *db/db* mouse display increased phenylephrine (PhE) contraction compared to control arteries.** The figures show concentration-response curves to PhE. Data represent the mean ± SEM of the values of contraction (% of 120 mM KCl-induced contraction). (*n* = 5–8 per group), *t*-test, *p < 0.05 vs. control.

**FIGURE 2:**
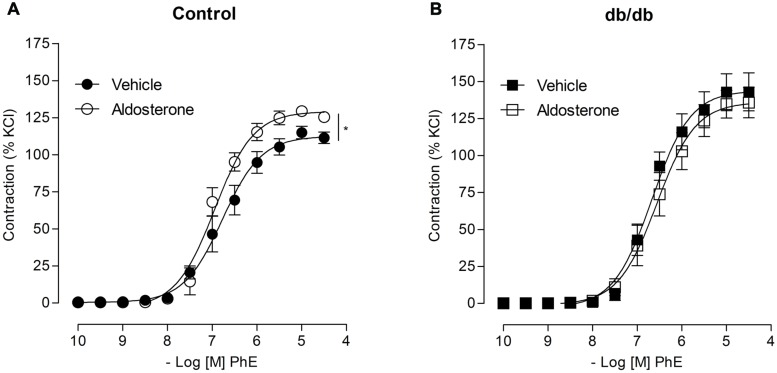
**Aldosterone increases PhE contractions in arteries from control, but not from *db/db* mice.** The figures show concentration–response curves to PhE in the presence of aldosterone (10 nM) in mesenteric arteries (MA) from control **(A)** and diabetic **(B)** mice. Data represent the mean ± SEM of the values of contraction (% of 120 mM KCl-induced contraction). (*n* = 5–7 per group), *t*-test, *p < 0.05 vs. vehicle.

Concentration–response curves to PhE were compared in arteries from spontaneously diabetic (*db/db*) and control mice. In the absence of any stimulus, arteries from *db/db* mice showed greater PhE-induced maximal contraction than control arteries (**Figure 1**). Incubation with aldosterone significantly increased maximal PhE-induced contraction (% of 120 mM KCl) in MA from control [**Figure 2A**; **Table 1** (*E*_max_ Control–vehicle: 114.9 ± 4.2%; aldosterone: 129.4 ± 2.2%, *p* < 0.05)], but not in arteries from *db/db* mice [**Figure 2B**; **Table 1** (*E*_max_ *db/db*–vehicle: 142.8 ± 12.5%; aldosterone: 134.7 ± 9.4%, *p* > 0.05)].

**Table 1:** Maximal responses to phenylephrine (PhE) in mesenteric arteries (MA) from control and *db/db* mice incubated with vehicle or aldosterone.

Groups	Control	*db/db*
Vehicle	114.9 ± 4.2 (*n* = 7)	142.8 ± 12.5 (*n* = 6)
Aldosterone	129.4 ± 2.2 (*n* = 5)*	134.7 ± 9.4 (*n* = 7)

The values show % of 120 mM KCl-induced contraction. The data represent the mean ± SEM of *n* experiments. *t*-test, *p < 0.05 vs. Vehicle.

### THE EFFECTS OF ALDOSTERONE ON PhE-INDUCED VASOCONSTRICTION DEPEND ON GPER ACTIVATION IN CONTROL MICE AND ON GPER AND MR ACTIVATION IN *db*/*db* MICE

**FIGURE 3:**
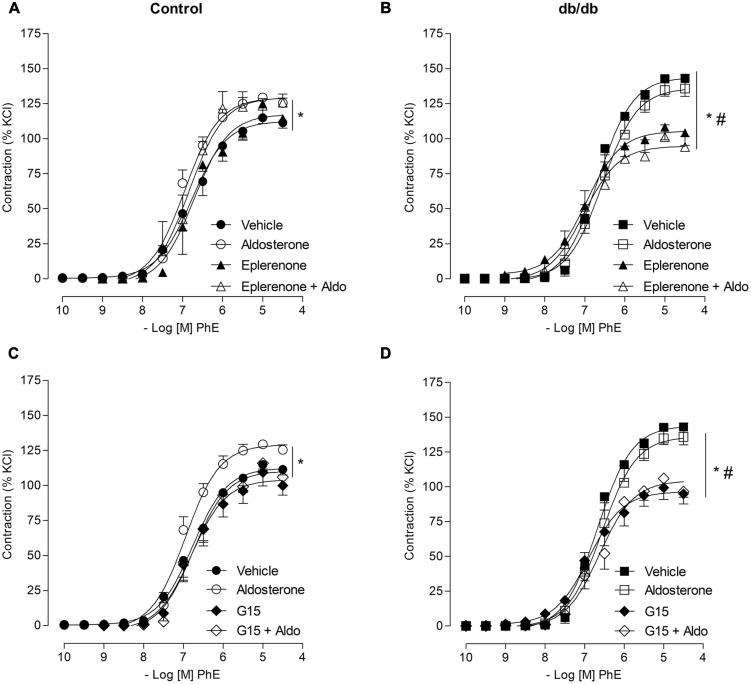
**Aldosterone increases PhE contraction in arteries from control mice through G protein-coupled estrogen receptors (GPER) activation, and by mechanisms that involve GPER and mineralocorticoid receptors (MR) activation in arteries from *db/db* mice.** The figures show concentration–response curves to PhE in the presence of aldosterone (aldo, 10 nM) in MA from control **(A,C)** and diabetic **(B,D)** mice. Effects of eplerenone (10 μM; **A,B**) or G15 (1 μM; **C,D**) on PhE-induced contraction in the absence and presence of aldosterone. Data represent the mean ± SEM of the values of contraction (% of 120 mM KCl-induced contraction). (*n* = 5–7 per group). One-way ANOVA with Bonferroni post-test (*), (#; *p* < 0.05) vs. vehicle and aldosterone, respectively.

In MA from the control group, pharmacological blockade of MR with eplerenone did not affect the effects of aldosterone on PhE-induced maximal contraction [**Figure 3A**; **Table 2**; (*E*_max_; Control–vehicle: 114.9 ± 4.2%; aldosterone: 129.4 ± 2.2%; eplerenone: 124.9 ± 2.2%; eplerenone + aldosterone: 123.5 ± 5.0%, *p* > 0.05)]. However, in the diabetic group, the increased vascular contraction to PhE was abolished in the presence of eplerenone, returning PhE-induced contraction to levels observed in control arteries (*E*_max_; *db/db*–vehicle: 142.8 ± 12.5%; aldosterone: 134.7 ± 9.4%; eplerenone: 97.9 ± 8.7%; eplerenone + aldosterone: 101.1 ± 8.8%, *p* < 0.05, **Figure 3B**; **Table 2**), and suggesting a role for the MR in the increased maximal vasoconstriction to PhE in *db/db* mice. The effects of aldosterone were abolished in the presence of G15, a GPER antagonist, in MA from control mice (*E*_max_; Control–vehicle: 114.9 ± 4.2%; aldosterone: 129.4 ± 2.2%; G15: 118.0 ± 5.8%; G15 + aldosterone: 105.9 ± 2.1, *p* < 0.05, when compare vehicle vs. aldosterone, aldosterone vs. G15 and aldosterone vs. G15 + aldosterone, **Figure 3C**; **Table 2**). In the diabetic group, G15 also decreased PhE maximal contraction (*E*_max_; *db/db*–vehicle: 142.8 ± 12.5%; aldosterone: 134.7 ± 9.4%; G15: 99.3 ± 8.5%; G15 + aldosterone: 98.6 ± 8.6%, *p* < 0.05, when compare aldosterone vs. G15 and aldosterone vs. G15 + aldosterone, **Figure 3D**; **Table 2**). These results demonstrate that GPER accounts for the effects of aldosterone on PhE maximal contraction in arteries from both groups.

**Table 2:** Maximal responses to PhE in MA from control and *db/db* mice incubated with vehicle or aldosterone in the presence or absence of mineralocorticoid receptors (MR) and G protein-coupled estrogen receptors (GPER) antagonists, eplerenone, and G15, respectively.

Groups	Control	*db/db*
Vehicle	112.5 ± 3.2 (*n* = 7)	142.8 ± 12.5 (*n* = 6)
Aldosterone (10 nM)	129.1 ± 2.8 (*n* = 5)*	134.7 ± 9.4 (*n* = 7)
Eplerenone (10 μM)	117.3 ± 4.6 (*n* = 3)#	97.9 ± 8.7 (*n* = 7)*#
Eplerenone (10 μM) + aldosterone (10 nM)	129.3 ± 5.5 (*n* = 4)*	101.1 ± 8.8 (*n* = 7)*#
G15 (1 μM)	104.1 ± 4.5 (*n* = 4)*	99.3 ± 8.5 (*n* = 6)*#
G15(1 μM) + aldosterone (10 nM)	110.3 ± 3.6 (*n* = 4)#	98.6 ± 8.6 (*n* = 6)*#

The data represent the mean ± SEM of *n* experiments. The values show % of 120 mM KCl-induced contraction. One-way ANOVA with Bonferroni post-test (*p* < 0.05). (*), (#) vs. Vehicle and aldosterone, respectively.

### ALDOSTERONE DECREASES ACh-INDUCED VASODILATION

**FIGURE 4:**
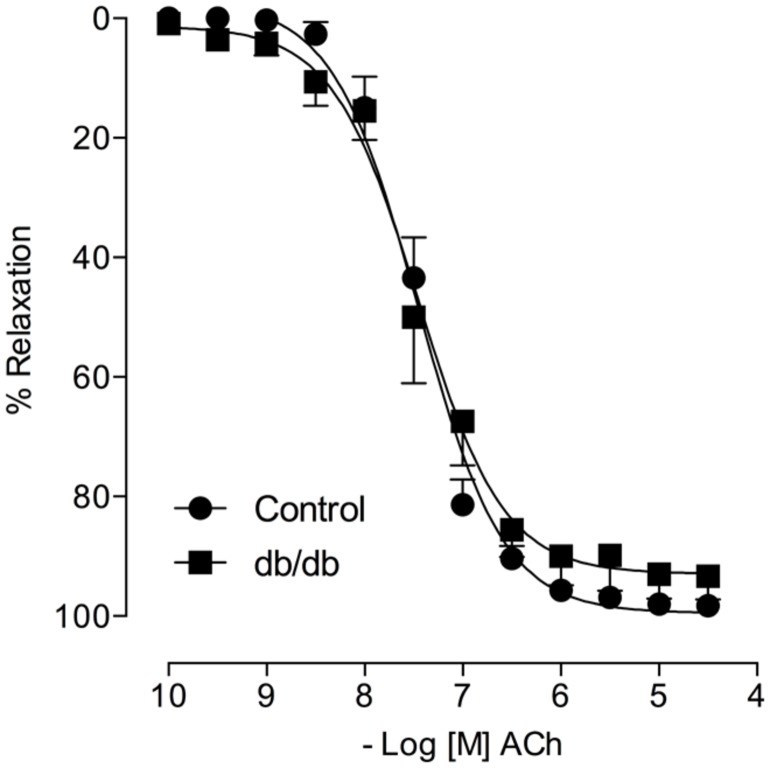
**Acetylcholine (ACh)-induced vasodilatation is similar in control and *db/db* mice.** The figures show concentration–response curves to ACh. Data represent the mean ± SEM. of the values of relaxation (% of PhE-induced precontraction). (*n* = 6–7 per group), *t*-test.

**FIGURE 5:**
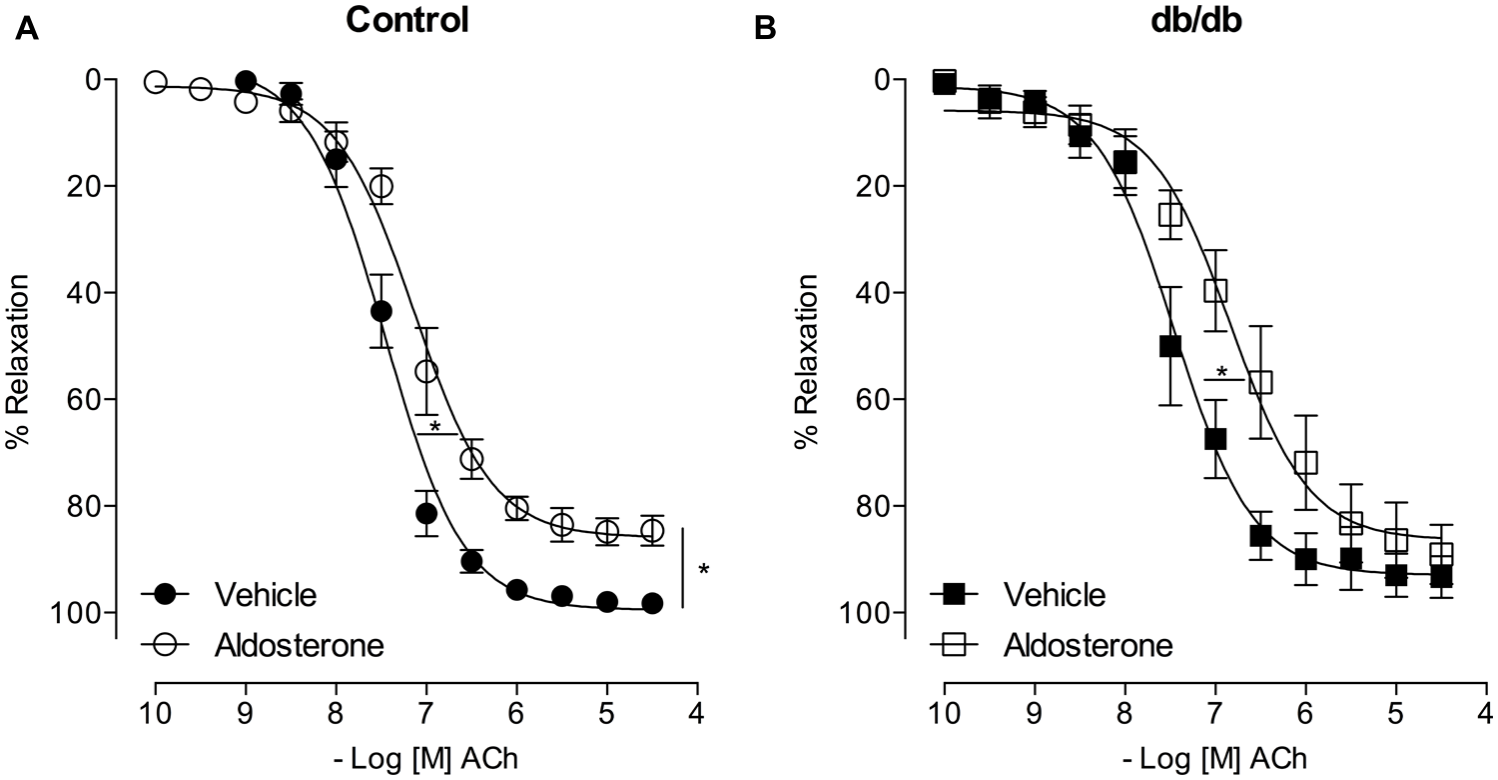
**Aldosterone decreases ACh potency (or ACh-induced relaxation) in control and *db/db* mice.** The figures show concentration–response curves to ACh in the presence of aldosterone (10 nM) in MA from control **(A)** and *db/db* **(B)** mice. Data represent the mean ± SEM of the values of relaxation (% of PhE-induced precontraction). (*n* = 5–7 per group). *t*-test, *p < 0.05 vs. vehicle.

Acetylcholine induced a similar concentration-dependent vasodilatation in MA from control and *db/db* mice (**Figure 4**). Aldosterone significantly decreased ACh maximal relaxation and potency in arteries from control mice (*E*_max_; Control–vehicle: 98.25 ± 0.7%; aldosterone: 84.6 ± 2.7%, *p* < 0.05, *p*D_2_; Control–vehicle: 7.5 ± 0.04; aldosterone: 7.1 ± 0.06, *p* < 0.05, **Figure 5A**, **Tables 3** and **4**). Aldosterone also decreased ACh potency in arteries from *db/db* mice (*E*_max_; *db/db*–vehicle: 93.3 ± 3.8%; aldosterone: 89.1 ± 5.6%, *p* > 0.05, *p*D_2_; *db/db*–vehicle: 7.4 ± 0.08; aldosterone: 6.8 ± 0.11, *p* < 0.05, **Figure 5B**).

**Table 3:** *p*D_**2**_ values of acetylcholine (ACh)-induced relaxation in MA of diabetic and control mice incubated with vehicle or aldosterone.

Groups	Control	*db/db*
Vehicle	7.46 ± 0.04 (*n* = 7)	7.45 ± 0.08 (*n* = 6)
Aldosterone (10 nM)	7.14 ± 0.06 (*n* = 5)*	6.83 ± 0.11 (*n* = 5)*
Eplerenone (10 μM)	6.88 ± 0.09 (*n* = 4)*	6.77 ± 0.11 (*n* = 5)*
Eplerenone (10 μM) + Aldosterone (10 nM)	7.13 ± 0.07 (*n* = 5)*	7.14 ± 0.12 (*n* = 5)
G15 (1 μM)	7.47 ± 0.09 (*n* = 5)#	7.23 ± 0.13 (*n* = 5)
G15 (1 μM) + Aldosterone (10 nM)	6.85 ± 0.08 (*n* = 4)*&	6.81 ± 0.10 (*n* = 5)*

The data represent the mean ± SEM of *n* experiments. One-way ANOVA with Bonferroni post-test (*p* < 0.05). (*), (#), and (&) vs. Vehicle, aldosterone and G15, respectively.

**Table 4:** Maximal responses to ACh-induced relaxation in MA from control and *db/db* mice incubated with vehicle or aldosterone in the presence or absence of MR and GPER antagonists, eplerenone and G15, respectively.

Groups	Control	*db/db*
Vehicle	98.25 ± 0.7 *(n =* 7)	93.3 ± 3.8 (*n* = 6)
Aldosterone (10 nM)	84.6 ± 2.7 (*n* = 5) *	89.1 ± 5.5 (*n* = 5)*
Eplerenone (10 μM)	90.9 ± 5.0 (*n* = 4)	87.9 ± 0.6 (*n* = 5)*
Eplerenone (10 μM) + aldosterone (10 nM)	96.4 ± 0.6 (*n* = 5) #	87.7 ± 7.4 (*n* = 5)
G15 (1 μM)	93.0 ± 1.1 (*n* = 5)	76.5 ± 7.3 (*n* = 5)
G15 (1 μM) + aldosterone (10 nM)	87.8 ± 6.0 (*n* = 4)	82.2 ± 5.8 (*n* = 5)*

The values show % of PhE-induced pre-contraction. The data represent the mean ± SEM of *n* experiments. One-way ANOVA with Bonferroni post-test (*p* < 0.05). (*), (#) vs. Vehicle and aldosterone, respectively.

### THE VASCULAR EFFECTS OF ALDOSTERONE ON ACh VASODILATATION DEPEND ON MR ACTIVATION IN CONTROL AND *db*/*db* MICE

**FIGURE 6:**
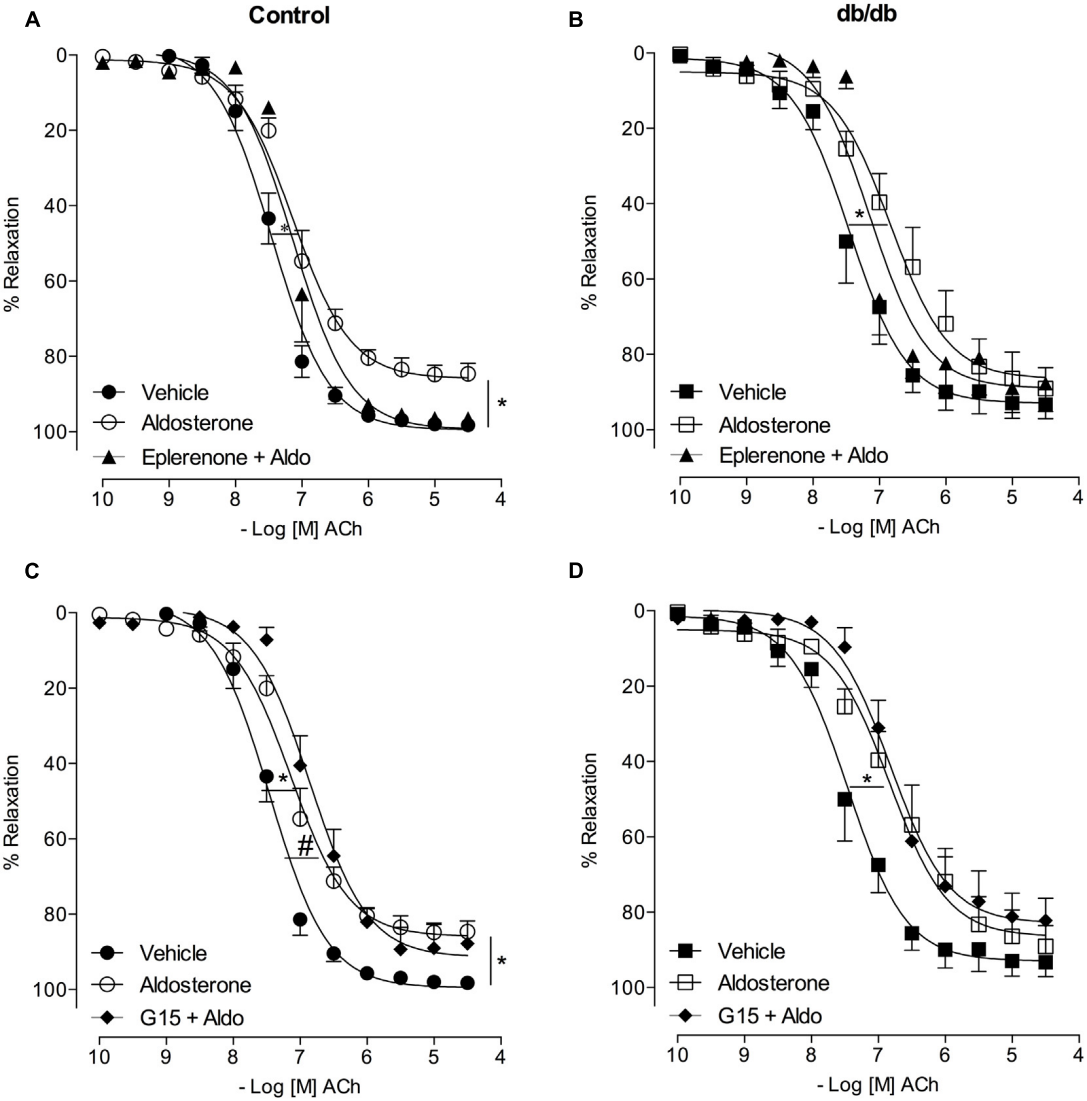
**Aldosterone decreases ACh-induced relaxation in control and *db/db* mice by mechanisms dependent on MR activation.** The figures show concentration-response curves to ACh in the presence of aldosterone (10 nM) in MA from control **(A,C)** and diabetic **(B,D)** mice. Effects of eplerenone (10 μM; **A,B**) or G15 (1 μM; **C,D**) on ACh-induced relaxation in the presence of aldosterone. Data represent the mean ± SEM of the values relaxation (% of PhE-induced precontraction). (*n* = 4–7 per group). One-way ANOVA with Bonferroni post-test (*), (#; *p* < 0.05) vs. vehicle and aldosterone, respectively.

In MA of the control group, eplerenone reversed the effects of aldosterone on ACh responses, returning ACh vasodilatation to values observed in basal conditions (*E*_max_; Control–vehicle: 98.2 ± 0.7%; aldosterone: 84.6 ± 2.7%; eplerenone + aldosterone: 96.4 ± 0.6%, *p* > 0.05, *p*D_2_; Control–vehicle: 7.5 ± 0.04; aldosterone: 7.1 ± 0.06; eplerenone + aldosterone: 7.1 ± 0.07, *p* < 0.05 when compare vehicle vs. aldosterone and aldosterone vs. eplerenone + aldosterone, **Figure 6A**; **Tables 3** and **4**). For the sake of clarity and to facilitate graphic interpretation, the effects of the antagonists alone were not displayed (unless they modified a specific response). In the diabetic group, the effects of aldosterone were partially reversed in the presence of eplerenone (*E*_max_; *db/db*–vehicle: 93.3 ± 3.8%; aldosterone: 89.1 ± 5.6%; eplerenone + aldosterone: 87.7 ± 7.4%, *p* > 0.05, *p*D_2;_ *db/db*–vehicle: 7.4 ± 0.08; aldosterone: 6.8 ± 0.11; eplerenone + aldosterone: 7.14 ± 0.11, *p* < 0.05 when compare vehicle *vs*. aldosterone and vehicle vs. eplerenone + aldosterone, **Figure 6B**; **Tables 3** and **4**). GPER antagonism further decreased ACh-induced relaxation in the control group (*E*_max_; Control–vehicle: 98.2 ± 0.7%; aldosterone: 84.6 ± 2.7%; G15 + aldosterone: 87.8 ± 6.0%, *p* < 0.05, *p*D_2_; Control–vehicle: 7.5 ± 0.04; aldosterone: 7.1 ± 0.06; G15 + aldosterone: 6.8 ± 0.07, *p* < 0.05 when compare vehicle vs. aldosterone, vehicle vs. G15 + aldosterone, and aldosterone vs. G15 + aldosterone **Figure 6C**; **Tables 3** and **4**) but did not affect ACh responses in the diabetic group (*E*_max_; *db/db*–vehicle: 93.3 ± 3.8%; aldosterone: 89.1 ± 5.6%; G15: 76.5 ± 7.3%; G15 + aldosterone: 82.2 ± 5.8%, *p* < 0.05, *p*D_2_; *db/db*–vehicle: 7.4 ± 0.08; aldosterone: 6.8 ± 0.11; G15 + aldosterone: 6.8 ± 0.09, *p* < 0.05 when compare vehicle *vs*. aldosterone and vehicle vs. G15 + aldosterone, **Figure 6D**; **Tables 3** and **4**). These results demonstrate that while the MR contributes to the effects of aldosterone on ACh relaxation, GPER is a counter-regulator of this deleterious effect of aldosterone in control, but in *db/db* mice.

### VASCULAR EFFECTS OF ALDOSTERONE AND ITS RECEPTORS WERE ASSOCIATED WITH GREATER ERK1/2 PHOSPHORYLATION

**FIGURE 7:**
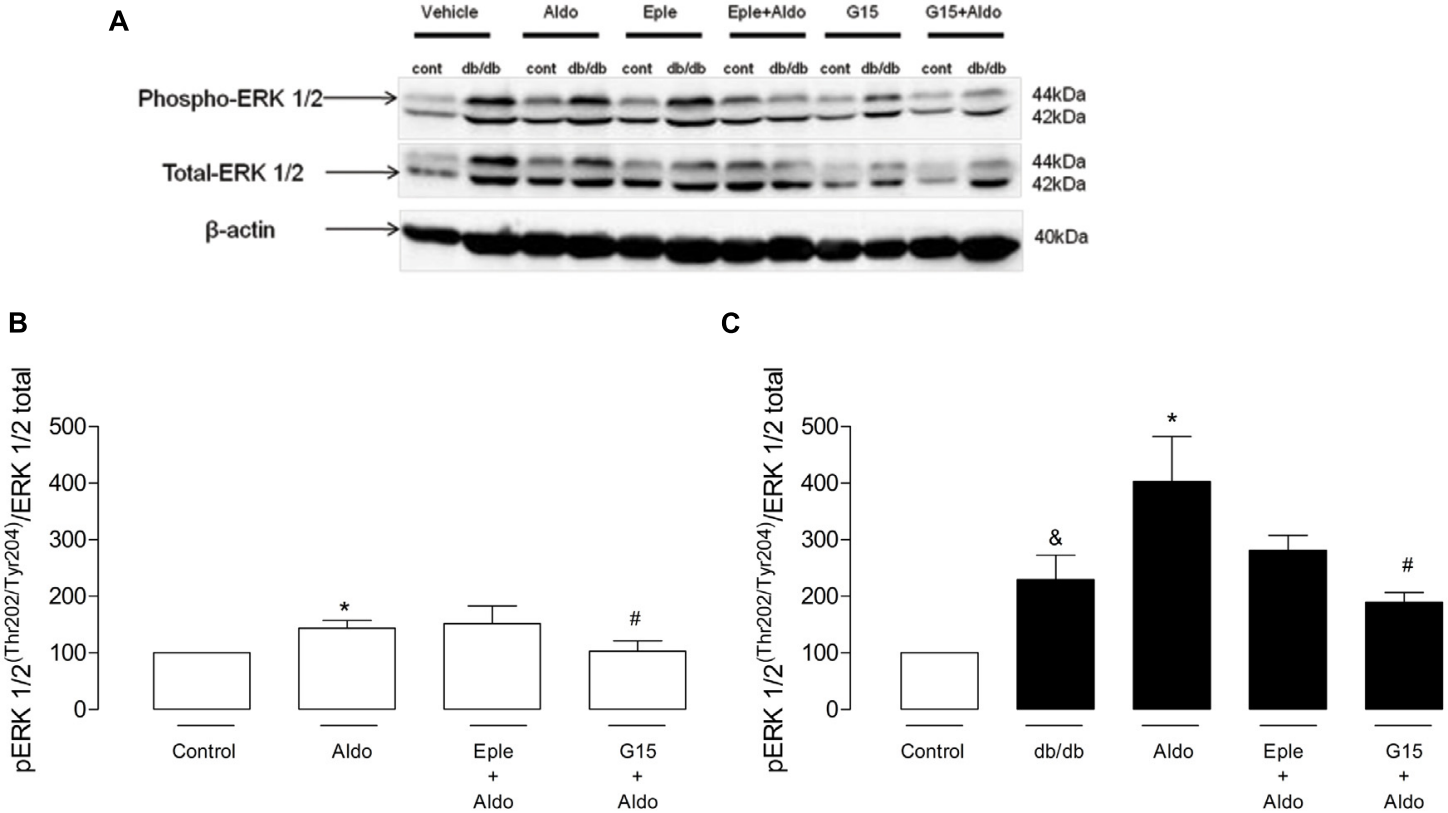
**Aldosterone increases vascular ERK 1/2 phosphorylation in control and *db/db* mice. (A)** Representative blots of phospho-ERK1/2, total ERK1/2, and b-actin. The effects of G15 (1 μM) and eplerenone (10 μM) on aldosterone (10 nM)-stimulated ERK1/2 phosphorylation in MA in control **(B)** and *db/db* **(C)** mice are also shown. Data represent the mean ± SEM from three independent experiments. (∗), (&), and (#) (*p* < 0.05) vs. control, control aldosterone, and *db/db* aldosterone, respectively.

To gain insight about the different role of MR and GPER on aldosterone-induced vascular functional changes, we investigated the involvement of ERK-1/2 pathway. *Db/db* mice showed greater expression of the phosphorylated form of ERK1/2 (pERK1/2) in the MA (Au, control: 99.8 ± 0.4 vs. *db/db*: 229.4 ± 42.9, *p* < 0.05, **Figure 7**), and aldosterone incubation further increased pERK1/2 expression in control arteries (Au, vehicle: 99.8 ± 0.4 vs. Aldo: 143.5 ± 13.7, *p* < 0.05) and *db/db* mice (Au, vehicle: 229.4 ± 42.9 vs. Aldo: 401.9 ± 80.2, *p* < 0.05, **Figure 7A**). Aldosterone-induced EKR1/2 phosphorylation was significantly reduced in control arteries treated with G15 (Au, Aldo: 143.5 ± 13.7 vs. G15 + aldo: 103.0 ± 18.5, *p* < 0.05), but not in arteries treated with eplerenone (Au, Aldo: 143.5 ± 13.7 vs. eplerenone + aldo: 151.5 ± 31.3, *p* < 0.05, **Figure 7**). In *db/db* arteries, both eplerenone and G15 reduced aldosterone-induced expression of pERK1/2 (Au, Aldo: 401.9 ± 80.2 vs. Eplerenone + aldo: 281.2 ± 26.5 vs. G15 + aldo: 189.4 ± 17.1, *p* < 0.05, **Figure 7B**).

### MR AND GPER EXPRESSION AND IMMUNOLOCALIZATION IN MA

**FIGURE 8:**
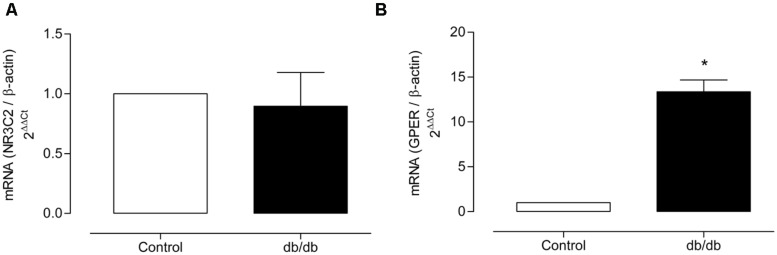
**Mineralocorticoid receptors and GPER mRNA expression.** The figure shows the values of 2^-ΔΔCt^ for MR **(A)** and GPER **(B)** in MA from the control and diabetic groups. The values were normalized by the corresponding β-actin expression. The data represent the mean ± SEM (*n* = 3–5 for each group). *t*-test, *p < 0.05 vs. control.

**FIGURE 9:**
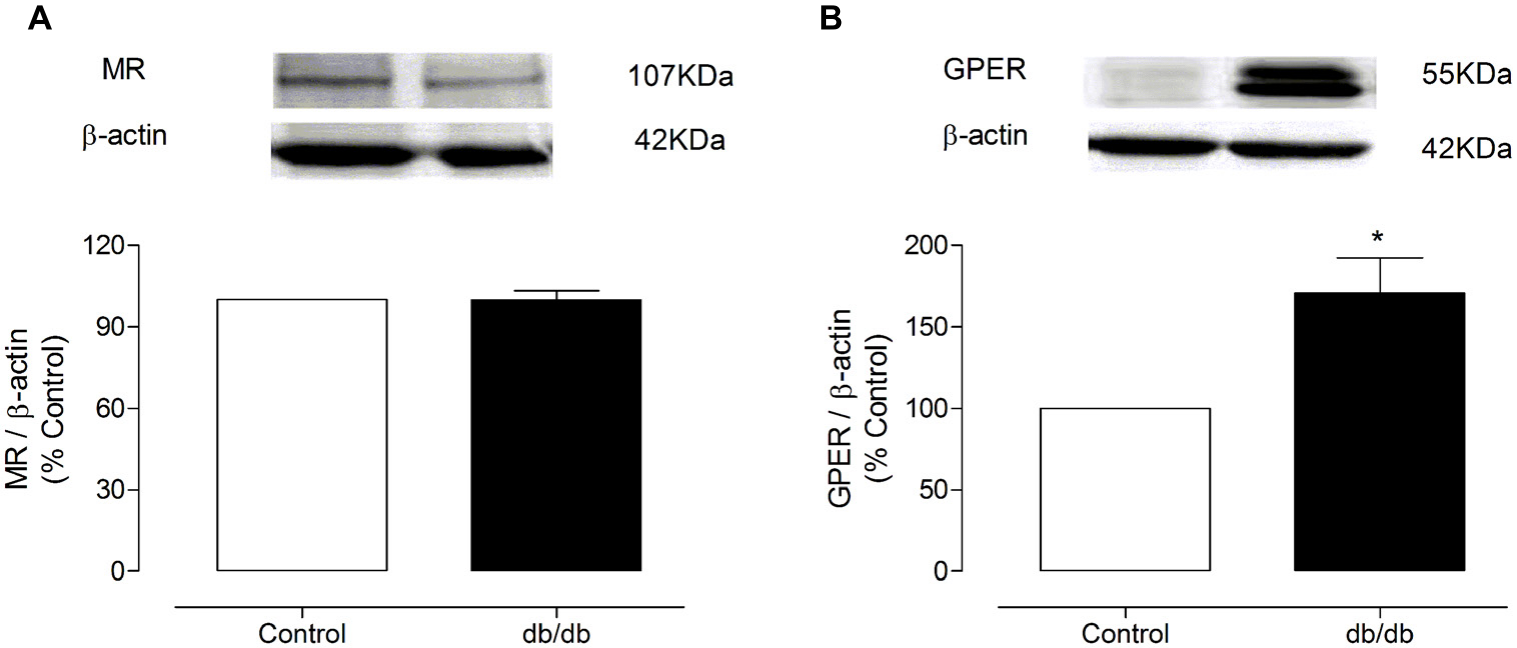
**Representative immunoblots for MR and GPER protein expression in MA from control and *db/db* mice.** The graph shows the MR **(A)** or GPER **(B)** protein levels, which were normalized to β-actin (MR or GPER/β-actin ratio). The data represent the mean ± SEM (*n* = 3–4 for each group). *t*-test, *p < 0.05 vs. control.

**FIGURE 10:**
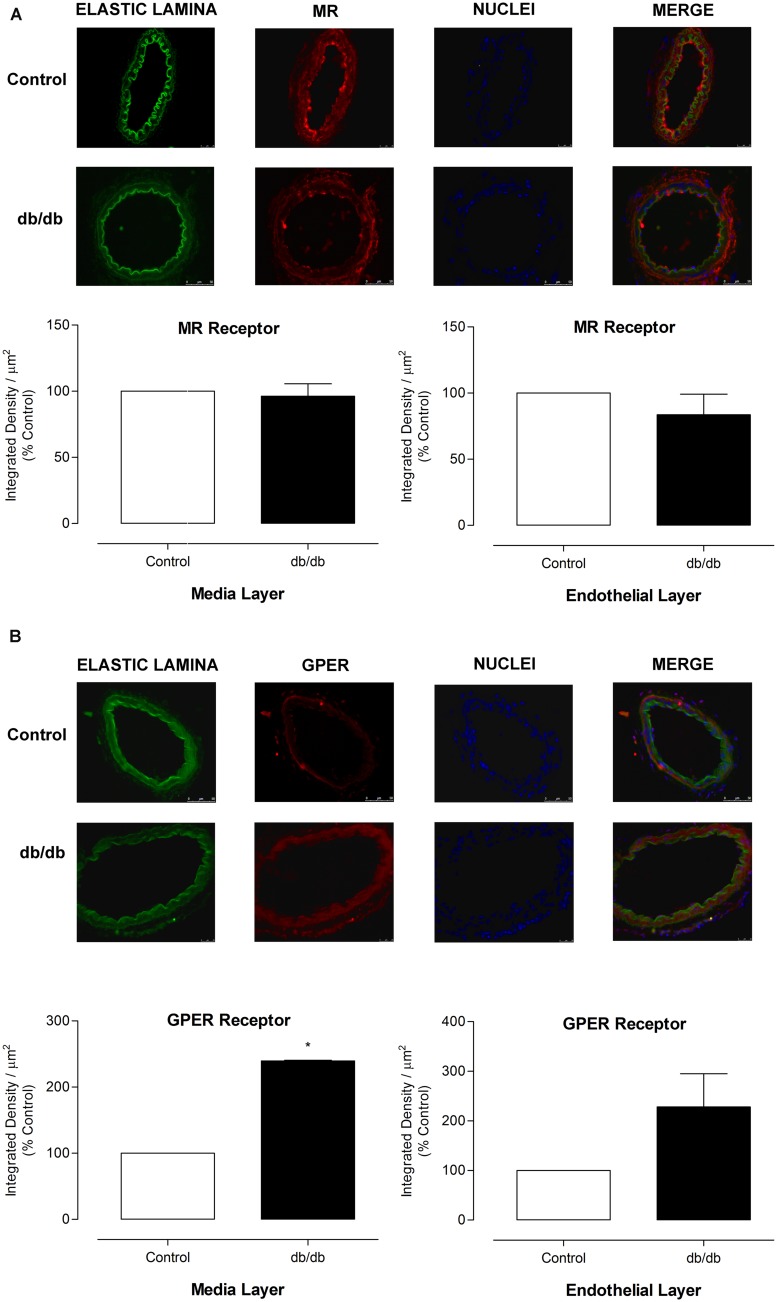
**Expression of MR and GPER in MA from control and *db/db* mice.** Representative photomicrographs (400x) and corresponding graphics of MR **(A)** and GPER **(B)** in MA from control and *db/db* mice. Graphics on the left depict expression of receptors in the media layer and on the right, the receptors expression in the endothelial layer. 4′,6-diamidino-2-phenylindole (DAPI, blue) was used to stain the nuclei. The elastic lamina autofluorescence is shown in green. The data represent the mean ± SEM for MR **(A)** or GPER **(B)** immunofluorescence (*n* = 3–6 per group). *t*-test, *p < 0.05 vs. control.

The mRNA expression of MR and GPER was determined by quantitative real-time PCR. Vascular mRNA expression of MR was similar between arteries from control and diabetic mice (**Figure 8A**, *p* > 0.05). On the other hand, MA from *db/db* mice displayed a significant increase of GPER mRNA expression compared to control arteries (**Figure 8B**, *p* < 0.05). Similar results were observed in the analysis of MR and GPER protein expression in MA, as shown in **Figure 9**. The localization of GPER and MR receptors in MA from control and *db/db* mice was determined by immunofluorescence staining in the media and endothelial layers. In accordance with results from the mRNA and immunoblotting assays, MR fluorescence intensity was similar in both groups (**Figure 10A**), while an increased GPER fluorescence was found in the media layer in vessels from *db/db* mice (**Figure 10B**). Immunolocalization of MR and GPER in the endothelial layer were similar in both groups.

## DISCUSSION

The present study shows that GPER differentially contributes to the vascular effects of aldosterone in control and diabetic conditions. Whereas MR mediates aldosterone-induced decreased ACh relaxation in MA from control and diabetic mice, GPER activation counter-regulates aldosterone effects in control, but not in *db/db* mice. To our knowledge, this is the first demonstration that aldosterone-induced reduced vasodilation is associated with loss of GPER protective effects in diabetes. Aldosterone also increases PhE contraction and ERK1/2 phosphorylation in MA via GPER activation in control mice and by GPER and MR-dependent mechanisms in arteries from *db/db* mice. Of importance, antagonism of both MR and GPER reverses vascular dysfunction, i.e., increased vascular responses to PhE, in diabetic mice, suggesting that intrinsic activation of both MR and GPER contributes to diabetes-associated increased vascular reactivity.

Aldosterone levels are increased in animal models of diabetes (Briones et al., 2012; Jin et al., 2013) as well as in diabetic patients (Hollenberg et al., 2004; Szymanski et al., 2011), and a role for aldosterone on diabetes-associated vasculopathies has been suggested and investigated (McFarlane and Sowers, 2003; Whaley-Connell and Sowers, 2011; Bender et al., 2013). In normal conditions, aldosterone acutely produces opposing effects on vascular reactivity. It induces nitric oxide (NO) generation and endothelium-dependent vasodilatation and also increases vascular smooth muscle cell reactive oxygen species (ROS) generation and vasoconstrictor responses, as revised by Feldam and Gros (Leopold, 2009; Feldman and Gros, 2013). The rational explanation for these discrepant effects has been based on the activation of MR located in different cells and/or the activation of different receptors, MR and/or GPER.

It is generally accepted that the deleterious effects of aldosterone (e.g., endothelial dysfunction, increased reactivity to constrictor stimuli, pro-oxidative, and pro-inflammatory effects) are mediated by MR activation. Accordingly, treatment with MR antagonists improves vascular function in animal models of diabetes and in diabetic patients (Schiffrin, 2006; Adel et al., 2014; Garg et al., 2014). On the other hand, by inducing endothelium-dependent and endothelium-independent vasodilatation (Yu et al., 2011), GPER activation, has been associated with vascular protective effects (Haas et al., 2007; Lindsey et al., 2011; Gros et al., 2013; Feldman and Gros, 2013; Lindsey et al., 2013).

Considering the vascular protective effects associated with GPER activation (Gros et al., 2013), and that aldosterone has been shown to activate GPER (Gros et al., 2011) we hypothesized that the beneficial effects mediated by vascular GPER activation are decreased in diabetes. Accordingly, aldosterone decreased ACh response in arteries from control mice and blockade of GPER further increased aldosterone-induced impairment of ACh vasodilation, indicating that GPER activation is important to counteract effects of aldosterone on endothelial cells. However, GPER antagonism did not interfere with acute aldosterone effects on endothelial function in *db/db* mice. Although expression of GPER was increased in MA from *db/db* mice, functional responses mediated by GPER activation were blunted. Of importance, our study was performed in resistance arteries, which actively contribute to peripheral resistance control (Christensen and Mulvany, 2001), and are also involved in diabetes-associated vasculopathies.

Our data showing that aldosterone increases PhE-induced vasoconstriction and vascular ERK1/2 phosphorylation via GPER activation in control mice and by GPER and MR-dependent mechanisms in arteries from *db/db* mice, further confirm a differential contribution of MR and GPER to aldosterone vascular effects in control and diabetic conditions. ERK1/2 activation is involved in the regulation of essential cellular processes, including gene expression, vascular smooth muscle cell proliferation, and contraction (Adam et al., 1995; Katoch and Moreland, 1995). Aldosterone rapidly induces ERK1/2 phosphorylation in smooth muscle and endothelial cells *in vitro* via activation of both MR and GPER (Gros et al., 2011). *In vivo*, the pathways involved in the effects of aldosterone seem to be more complex and dependent on the vascular bed studied. For instance, aldosterone increases angiotensin II (Ang-II)-induced vasoconstriction in human coronary micro arteries by GPER- and EGFR-dependent mechanisms, but independently of ERK1/2 phosphorylation. In addition, aldosterone regulates apoptosis and activation of MLC, a contraction-promoting protein, in GPER-enriched vascular smooth muscle cells (Gros et al., 2011), unlinking the classical view of MR as the major mediator of aldosterone-induced vascular damage.

Other studies have shown that activation of GPER by its agonist G1 promotes endothelium-dependent and -independent relaxation (Lindsey et al., 2011; Yu et al., 2011), and chronic treatment with G1 reverses vascular dysfunction in aorta from diabetic female rats (Li et al., 2012). Together these results suggest that diabetes-associated vascular dysfunction is partially associated with decreased GPER activity.

It was recently demonstrated that GPER is expressed in adult human adrenal cortex and in aldosterone-producing adenoma cells (Caroccia et al., 2014). Curiously, aldosterone is locally synthesized in resistance arteries by the perivascular adipose tissue (PVAT; Briones et al., 2012). Therefore, it is possible that GPER influences the local production of aldosterone in the PVAT and, consequently, influences vascular contraction. This would explain the decrease of PhE-responses in *db/db* arteries exposed to MR and GPER antagonists, in a condition where no exogenous aldosterone was added. Nevertheless, this hypothesis remains untested.

Our study has limitations that should be pointed out: (1) it does not show a direct molecular interaction between aldosterone and GPER. This has been questioned in the literature since recent data show that aldosterone does not inhibit ^3^H-estradiol binding in kidney mice or HEK293 cells (Cheng et al., 2014; Prossnitz and Barton, 2014). (2) It does not explain the differential GPER-mediated responses in arteries of diabetic animals (blunted GPER responses to counteract aldosterone effects on ACh vasodilation vs. a contribution of GPER to aldosterone-induced greater contractile responses and ERK1/2 activation in arteries of *db/db* mice). Although one possible explanation for these discrepant effects would be that diabetes is associated with a specific loss of GPER-mediated effects on endothelial cells, with maintained GPER-mediated effects on smooth muscle cells, this remains to be tested.

In the present study we used female mice. Females are more susceptible to diabetes-associated cardiovascular complications (Toledo et al., 2003; Goel et al., 2007, 2008). GPER signaling induced by estrogen is protective, and it is lost in diabetic females. This effect is aggravated in menopause condition since premenopausal women have a lower risk of developing cardiovascular disease compared to men and postmenopausal women (Barrett-Connor and Wingard, 1983; Sowers, 1998; McCollum et al., 2005; Legato et al., 2006). Many studies with diabetic animals were conducted in ovariectomized rats aiming to demonstrate the effect of estrogen withdrawn on GPER activation. Responses elicited by androgens in *db/db* female mice were not fully comparable with those in ovariectomized female diabetic rats perhaps because *db/db* mice normally have low estrogen and high aldosterone levels (Garris, 1985; Garris et al., 1986; Jin et al., 2013), which can generate adaptations that are not known yet.

In summary, our results indicate that aldosterone differentially activates MR and GPER in arteries from control and diabetic mice. Activation of GPER by aldosterone triggers opposite effects on vascular reactivity and the final response depends on the balance between the vasodilator effects, usually mediated by the endothelium, and vasoconstrictor effects by actions on smooth muscle cells. In diabetes, where endothelial dysfunction is present, contractile effects prevail and the beneficial effects of GPER in the vasculature are decreased/lost, supporting our hypothesis that the beneficial vascular effects mediated by GPER activation are decreased in diabetes mellitus.

## CONCLUSION

The counter-regulatory (beneficial) effects of aldosterone mediated by GPER activation in the vasculature are decreased in diabetes mellitus, whereas vascular pro-contractile aldosterone effects are maintained. The mechanisms by which aldosterone impairs vascular function in diabetes may include a differential activation of MR and GPER in arteries from control and diabetic subjects. Our results contribute to understanding the mechanisms by which aldosterone influences vascular function and contributes to vascular damage in diabetes.

## AUTHOR CONTRIBUTIONS

NF, SC, MS, CM, and FM performed wet laboratory experiments. NF, SC, FC designed the study. NF, TM, FC, RT, wrote the manuscript and revised its scientific content.

## Conflict of Interest Statement

The results presented in this paper have not been published previously in whole or part, except in abstract format. The authors declare that the research was conducted in the absence of any commercial or financial relationships that could be construed as a potential conflict of interest.
